# Efficacy and safety of tuina for senile insomnia: A protocol for systematic review and meta-analysis

**DOI:** 10.1097/MD.0000000000028900

**Published:** 2022-02-25

**Authors:** Yangshengjie Liu, Xuejiao Bai, Hongshi Zhang, Xiaoyu Zhi, Jundong Jiao, Quanwu Wang, Yuanyuan Ji, Xu Zheng, Xinlu Zhang, Xue Tong, Jiayi Liu, Yahui Sun, Peng Liu

**Affiliations:** a Department of Acupuncture and Tuina, Changchun University of Chinese Medicine, Changchun, China; b Acupuncture and Massage center, The Third Affiliated Clinical Hospital of Changchun University of Chinese Medicine, Changchun, China; c Nursing College of Changchun University of Chinese Medicine, Changchun, China; d Department of Acupuncture and Tuina, The Affiliated Hospital of Changchun University of Chinese Medicine, Changchun, China; e Department of Tuina, Shenzhen Hospital of Guangzhou University of Chinese Medicine (Futian), Shenzhen, China.

**Keywords:** meta-analysis, protocol, senile insomnia, tuina

## Abstract

**Background:**

Insomnia is a common diseases of the elderly, tuina is a widely used treatment. At present, there is a lack of supportive evidence on efficacy and safety of tuina for senile insomnia. The purpose of this systematic review is to assess the effectiveness and safety of tuina therapy in the treatment of senile insomnia.

**Methods:**

Literature on tuina for senile insomnia in the PubMed, EMBASE, Web of Science, Cochrane, China National Knowledge Infrastructure Database, Wanfang, Chinese Scientific and Journal Database, Japanese medical database, Korean Robotics Institute Summer Scholars, and Thai-Journal Citation Index Center will be conducted to search from the creation of these databases. We will search the databases from the beginning to January 2022. The primary outcome was the Pittsburgh Sleep Quality Index score, and the secondary outcomes included clinical efficacy and safety. RevMan 5.4.1 will be used for the meta-analysis.

**Results:**

This study aimed to will prove the effectiveness and safety of tuina therapy for the treatment of insomnia in the elderly.

**Conclusion:**

This study provides up-to-date evidence of the effectiveness and safety of tuina for the treatment of senile insomnia.

**INPLASY registration number:**

INPLASY2021110063.

**Ethics and Communication:**

This systematic review will evaluate the effectiveness and safety of massage therapy for insomnia in the elderly population. As all the included data have been published, systematic reviews do not require ethical approval.

## 1 Introduction

Insomnia is a common disease among the elderly, and its clinical manifestations are insufficient sleep time, shallow sleep degree, and feeling of fatigue after waking up.^[1]^ Approximately 10% to percent of the world's population suffer from insomnia, and its incidence is increasing every year.^[2,3–7]^ Related studies show that insomnia in the elderly is related to cardiopathy, dementia, hypertension, cerebral hemorrhage, migraine, restless legs syndrome, and so on. Insomnia seriously affects the quality of life and physical and mental health of the elderly, but also increases the economic burden of the whole society and family.^[8]^ The treatment of insomnia mainly includes cognitive behavioral therapy and drug therapy.^[9–10]^ Benzodiazepines and non-benzodiazepines are mainly used for the pharmacological treatment of insomnia.^[11]^ Although drug treatment is effective,^[12–13]^ long-term use can lead to tolerance and dependence in patients, and^[14]^ older people are also at increased risk of falls. Therefore, there is an urgent need for a safer and more effective way to relieve insomnia. As a kind of complementary and alternative medicine,^[15]^ TCM external therapy is widely used in insomnia treatment, especially tuina, which has achieved good clinical effect. At present, there are no systematic reviews of massage therapy for insomnia in the elderly. This study aimed to evaluate the effectiveness and safety of massage therapy in elderly patients with insomnia, and provide a basis for clinical decision-making.

## 2 Methods and analysis

Our protocol should be based on the Preferred Reporting Items for Systematic Reviews and Meta-Analyses Protocols (PRISMA-P) statement guidelines.

Registered with INPLASY (INPLASY2021110063.)

### 2.1 Inclusion criteria

#### 2.1.1 Types of studies

Include all relevant randomized controlled trial using tuina for the treatment of senile insomnia.

#### 2.1.2 Types of patients

Senile patients with insomnia (≥60 years old), regardless of sex, race, condition, or intensity.

#### 2.1.3 Types of intervention

All randomized controlled trials of tuina therapy will be included, and the treatment group will be treated with tuina, while the control group will be treated with oral Chinese medicine, acupuncture, physiotherapy, and placebo, even without treatment.

#### 2.1.4 Outcomes

The primary outcome was the Pittsburgh Sleep Quality Index, and the secondary outcomes included clinical efficacy and safety.

### 2.2 Database search strategy

Literature on tuina for senile insomnia in the PubMed, EMBASE, Web of Science, Cochrane, China National Knowledge Infrastructure Database, Wanfang, Chinese Scientific and Journal Database (VIP), Japanese medical database), Korean Robotics Institute Summer Scholars, and Thailand Thai-Journal Citation Index Centre will be conducted to search from the creation of these databases. We will search the databases from the beginning to January, 2022. The search terms included senile insomnia, senile sleep disorder, senile sleep disturbance, tuina, massage, and massage therapy. The search strategy for PubMed is presented in Table 1.

**Table 1:** Search strategy for PubMed.

No	Search terms
#1	senile insomnia
#2	senile sleep disorder
#3	senile sleep disturbance
#4	or #1-#3
#5	Tuina
#6	massage
#7	massage therapy
#8	or #4-#6
#9	randomized controlled trial
#10	controlled clinical trial
#11	randomized
#12	randomly
#13	trial
#14	or #9-#13
#15	#4 and #8 and #14

### 2.3 Data collection and analysis

#### 2.3.1 Study selection and data extraction

**Figure 1:**
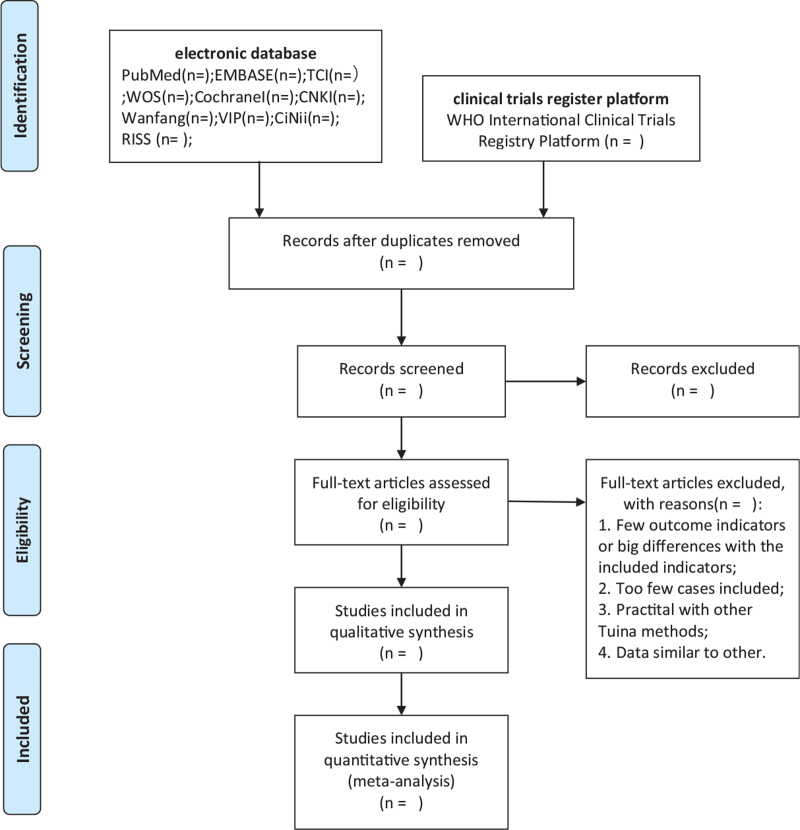
PRISMA flow diagram of study and exclusion. PRISMA = Preferred Reporting Items for Systematic Reviews and Meta analyses.

We imported the retrieved content to import the document into EndNote (V.X9.0) software to, delete duplicate data. Two researchers independently performed selection, data extraction, and quality assessment. After reading the article titles and abstracts, irrelevant literature was removed. Finally, articles to be included were selected by filtering the full articles. If disagreements arise during the evaluation process, the 2 reviewers will decide whether to discuss or consult a third reviewer. Data on the study design, participant characteristics, intervention, and control group intervention will be extracted and recorded in the electronic text. Extraction will be performed independently by two reviewers, and the information will be cross-checked. These differences will be discussed by a third author. The filtering process is illustrated in Figure 1.

#### 2.3.2 Assessment of the risk of bias and reporting of study quality

Two independent authors assessed the risk of bias using the Cochrane Bias Risk Tool. Each study was assessed as either high or low, and the risk of bias for each project was unclear. The differences will be resolved through further discussion with the third author. Complete STRICTA checklist. In addition, the Jadad scale was used to assess study quality.

#### 2.3.3 Measures of treatment effect

We will conduct statistical analysis the RevMan 5.4.1 software (Cochrane Training site based in London, UK). We using risk ratios and 95% confidence intervals for calculations as continuous data. Relative risk also represents dichotomous data.

#### 2.3.4 Units of analysis

The sleep quality values were summarized according to the results, and secondary outcomes, such as the safety and efficacy of the Pittsburgh Sleep Quality Score, total score of the Insomnia Severity Index, and changes in TCM symptoms, were analyzed separately.

#### 2.3.5 Management of missing data

When important information is missing, the corresponding author will be contacted by email or telephone, and if the information cannot be obtained through the preceding methods, the abnormal information will be deleted.

#### 2.3.6 Assessment of heterogeneity

We will use the Q-test and *I*^2^ statistic to assess statistical heterogeneity, *I*^2^ < 50% indicates insignificant heterogeneity, when *I*^2^≥50% indicates significant heterogeneity.

#### 2.3.7 Assessment of reporting bias

When more than 10 trials were included, a funnel plot was used to assess reported bias. Symmetry explains these deviations. If the funnel plot is symmetric, there was no bias, if it was asymmetric, there was bias.

#### 2.3.8 Data synthesis

Quantitative analysis will be performed using the RevMan 5.4.1 software with 95% confidence intervals. The average changes in each primary and secondary outcome were combined. Qualitative descriptions were used if the data were unsuitable for quantitative analysis.

#### 2.3.9 Subgroup analysis

If the clinical trials described above lead to heterogeneity, subgroup analyses will be performed based on interventions, different controls, duration of treatment, and outcome measures. We listed the adverse reactions and evaluated them.

#### 2.3.10 Sensitivity analysis

For quality analysis, we performed a sensitivity analysis of the main results to explore the influence of individual studies on the bias of the results.

## 3 Discussion

Sleep disorders are common in older adults and have been associated with^[16–18]^ nocturia, depression, and metabolic syndrome in addition to previously listed symptoms, age is thought to be the main cause of insomnia, and the incidence of insomnia increases significantly with age.^[19–20]^ Therefore, we will systematically review the curative effect of tuina in the treatment of insomnia in the elderly, which is conducive to the promotion and application of massage in the clinical treatment of insomnia in the elderly. We hope that this study will provide high-level evidence for the application of massage in the treatment of insomnia in the elderly population.

## Author contributions

**Conceptualization:** Yangshengjie Liu, Peng Liu.

**Data curation:** Yangshengjie Liu, Jiayi Liu, Yahui Sun, Peng Liu.

**Formal analysis:** Xiaoyu Zhi, Quanwu Wang, Yuanyuan Ji.

**Funding acquisition:** Jundong Jiao, Peng Liu.

**Investigation:** Xu Zheng, Xinlu Zhang, Xue Tong.

**Methodology:** Yangshengjie Liu, Hongshi Zhong.

**Supervision:** Peng Liu.

**Validation:** Yangshengjie Liu, Xuejiao Bai, Peng Liu.

**Writing – original draft:** Yangshengjie Liu.

**Writing – review & editing:** Yangshengjie Liu, Xuejiao Bai, Peng Liu.
